# Poor Sleep and Obesity: Concurrent Epidemics in Adolescent Youth

**DOI:** 10.3389/fendo.2018.00364

**Published:** 2018-07-10

**Authors:** Anisha Gohil, Tamara S. Hannon

**Affiliations:** ^1^Pediatric Endocrinology Fellow, Section of Pediatric Endocrinology/Diabetology, Riley Hospital for Children at IU Health, Indiana University School of Medicine, Indianapolis, IN, United States; ^2^Section of Pediatric Endocrinology/Diabetology, Riley Hospital for Children at IU Health, Indiana University School of Medicine, Indianapolis, IN, United States

**Keywords:** obesity, poor sleep, obstructive sleep apnea, sleep duration, sleep quality, diet quality, cardiometabolic risk, insulin sensitivity

## Abstract

Poor sleep and obesity are both extraordinarily common in the US adolescent population and often occur simultaneously. This review explores the links between obesity and sleep, outlining what is known about the relationships between sleep characteristics, obesity, and cardiometabolic risk factors in youth. Sleep duration is less than optimal in teens, and decreases as age increases. This is detrimental to overall well-being and is associated with obesity in children, adolescents, and young adults. Accordingly, inadequate sleep duration is associated with poor diet quality, decreased insulin sensitivity, hyperglycemia, and prevalent cardiometabolic risk factors. Evidence suggests that poor sleep quality and altered circadian timing characterized by a preferred later sleep onset, known as “adolescent chronotype,” contributes to shortened sleep duration. Obstructive sleep apnea (OSA) occurs more frequently among youth with obesity, and is associated with autonomic nervous system activity promoting higher blood pressure, increased markers of cardiovascular disease risk, and insulin resistance. While there is a clear association between OSA and type 2 diabetes in adults, whether or not this association is prevalent in youth is unclear at this time. Interventions to improve both sleep duration and quality, and obesity in adolescents are scarce and more evidence is needed to determine if such interventions can improve obesity-related health outcomes.

## Introduction

Data from the National Health and Nutrition Examination Survey (NHANES) shows the prevalence of obesity, defined as having a body mass index (BMI) at or above the 95th percentile for age and sex, in 2–19 year old youth was 16.8% in 2007–2008 and increased to 18.5% (nearly 1 in 5) in 2015–2016 (1). In addition, since 2013–2014, there has been a significant increase in obesity among youth ages 2–5 years, with an increase in prevalence from 9.3 to 13.7% in 2015–2016 (2). Thus, the burden of childhood obesity in the US continues to increase and as more youth are affected, co-morbid conditions are becoming more common (1).

Short sleep duration and poor sleep quality are also common in the pediatric population, especially in teens (3). Whether or not these two increasingly prevalent pediatric problems are physiologically linked is a topic of investigation and associations between sleep characteristics and obesity are beginning to be better understood. This review explores the links between obesity and sleep, outlining what is known about the relationships between sleep characteristics, obesity, and cardiometabolic risk factors in youth with a particular focus on sleep duration and obesity, and obstructive sleep apnea (OSA) and its metabolic consequences.

## Optimal sleep

It is clear that the majority of youth are sleeping less than is recommended for optimal health. In 2015 the National Sleep Foundation released recommendations for optimal sleep durations for healthy individuals (4). It is recommended that school-aged children, ages 6–13 years, should target 9–11 h of sleep per night, while adolescents, ages 14–17 years, should target 8–10 h of sleep per night. However, according to a 2006 survey conducted by the National Sleep Foundation, actual self-reported sleep times were significantly less than 9 h in all adolescent age groups and progressively declined from early to late adolescence (5). Adolescents ages 11–12 years reported a mean sleep duration of 8.4 h, while adolescents ages 17–18 years reported just 6.9 h of sleep per night. This shortened sleep duration was largely due to later bedtimes and the adolescents were self-aware of their deficient sleep. This is particularly important because adolescents are not meeting their physiological needs for restorative sleep. Adolescent “sleep need,” defined as the amount of sleep recorded when adolescents are given the opportunity to sleep for 10 h, is approximately 9 h and remains unchanged throughout adolescence (5).

## Sleep duration and obesity

Sleep duration has been associated with obesogenic behaviors and obesity prevalence in both adult and youth populations. A study of 240 toddlers from low-income families demonstrated that decreased mean sleep duration from 9.2 to 8.5 h was significantly associated with obesity (6). Moderate-to-vigorous physical activity, measured with an Actical accelerometer, was positively related to sleep duration. While sleep characteristics such as co-sleeping, room sharing, later sleep onset time, increased sleep latency, and night awakenings did not correlate with obesity, they were associated with decreased sleep duration and obesogenic behaviors including less physical activity and poorer diet quality.

The precise mechanisms linking rapid eye movement (REM) sleep and obesity are incompletely understood, but may include decreased sleeping metabolic rate, and endocrine changes associated with decreased leptin and increased ghrelin levels promoting increased food consumption (7). Sleep deprivation is associated with decreased insulin sensitivity via alterations of the hormonal milieu including cortisol, ghrelin, leptin, growth hormone, and glucose tolerance (8, 9). These hormonal changes cause alterations in energy regulation, unhealthy food choices, increased food consumption, decreased physical activity, and perhaps a reduction in non-exercise activity thermogenesis.

Decreased REM sleep is observed in individuals with short sleep duration, and has been a proposed mechanism for the link between short sleep duration and increased weight status. A study by Liu et al. involving 335 youth examined sleep stages using polysomnography and found significant differences in overweight children compared to normal-weight children, as they slept less, had reduced sleep efficiency, decreased REM sleep time, activity and density, and longer latency to the first REM episode (7). After adjustment for demographics, pubertal status, and confounding medical conditions, 1 h less of REM sleep was associated with an approximately 2-fold increased odds of being overweight, and 1 h less of REM sleep was associated with an approximately 3-fold increased odds of being overweight.

Leptin and ghrelin are key hormones involved in appetite regulation (10). In their review article, Van Cauter et al. discussed neuroendocrine control of food intake (11). In sleep-restricted as opposed to well-rested individuals, leptin levels are decreased while ghrelin levels are increased resulting in subjective hunger. The amount of sleep restriction varied among the reviewed studies. Much less is known about leptin and ghrelin secretion and operation in children. Recent studies have shown conflicting results. While one study showed short sleep duration was associated with lower leptin levels, the other revealed short sleep duration was associated with higher leptin levels (12). Table 1 summarizes some proposed mechanisms for poor sleep and obesity and some obesogenic behaviors seen in those with poor sleep.

**Table 1:** Proposed mechanisms and obesogenic behaviors in poor sleep and obesity.

**Proposed mechanisms**	**Obesogenic behaviors**
Evening Chronotype ◦ With early wake time ◦ Social jetlag Bedtime Shift Hormonal Alterations ◦ Leptin ◦ Ghrelin	Unhealthy Food Choice ◦ Sugar-sweetened beverages ◦ Fast food ◦ High calorie snacks Excessive portion sizes Increased food consumption Increased perceived hunger Food preoccupation

The associations between sleep duration and obesity are thought to be mediated, at least in part, by diet quality. Less sleep is consistently linked with unhealthy dietary habits including larger portion sizes, increased perceived hunger, higher calorie food choices, and increased food and sugar-sweetened beverage intake (13, 14). Cespedes et al. examined 1,046 parental reports of sleep and diet in children from infancy to mid-childhood (15). Sleep was measured using a calculated sleep score based on parental reported sleep duration. Diet was measured using the Youth Healthy Eating Index (YHEI). Chronic sleep insufficiency was associated with lower YHEI score. Looking independently at each factor, children with less sleep and a less healthy diet had a higher BMI z-score. It is not known whether or not these were causal associations.

A systematic review and meta-analysis of 17 observational, cohort, cross-sectional, and case-control studies from 9 countries found convincing evidence of the link between shorter sleep duration and childhood obesity (9). Sleep durations of less than 9 h (children 10 years of age or older), less than 10 h (children between 5 and 10 years of age), and less than 11 h (children less than 5 years of age) were associated with a 58% increased risk of being overweight or obese. Each hour increase in sleep duration was associated with a 9% reduced risk of being overweight or obese. Interestingly, the decreased sleep duration (defined by each studies' own criteria) and obesity association appeared to be stronger for boys than girls, with an odds ratio of 2.50 vs. 1.24 respectively (9), although the reason for this gender variance is not known and data from studies are conflicting (16–18). Cappuccio et al. published a meta-analysis reviewing the association between sleep duration and obesity in both youth and adults (19). Results in children correlated with previous findings (9), with an increased pooled odds of 1.89 for a shortened sleep duration associated with obesity (19). This association was also observed in adults from 12 countries and in various age groups from adolescence through late-adulthood, showing their results had a similar effect across populations and age groups.

Modifiable factors presumed to cause decreased sleep duration include evening exposure to electronic media, early school start times, academic workload, and caffeine consumption (3, 20). A systematic review of 33 studies to investigate the relationship between short sleep duration and potential mechanisms such as dietary habits, physical activity or lack thereof, screen time, and hormonal effects including, alterations in leptin and ghrelin levels, found evidence for associations of sedentary behavior, unhealthy dietary patterns, and insulin resistance with shorter sleep duration (8). Insulin resistance was assessed using one randomized controlled trial, one prospective cohort study, and one cross-sectional study, two with a negative association and one with a U-shaped association between sleep duration and HOMA-IR (homeostatic model assessment of insulin resistance). Conclusions for other mechanisms of physical activity, screen time, changes in leptin and ghrelin levels were indeterminate, attributed to lack of current evidence and need for more rigorous research.

## Sleep quality and obesity

Other indicators of sleep quality beside sleep duration have also been implicated as risk factors for obesity, although data are sparse. The National Sleep Foundation identified indicators of sleep quality for all age groups as: sleep onset latency, number of awakenings lasting less than 5 min, wake time after sleep onset, and sleep efficiency (ratio of total sleep time to time in bed) (21). Part of a cross sectional study by Quick et al. of 1,252 college students across nine U.S. universities evaluated sleep quality by self-report using the Pittsburgh Sleep Quality Index and weight status using two categories, normal weight (BMI less than 25) and overweight or obese (BMI 25 or greater) (22). Poorer sleep quality was significantly associated with being overweight or obese with an odds ratio of 1.07 (95% CI 1.01–1.13).

Fatima et al. published the first systematic review and meta-analysis looking at associations between sleep quality using the Pittsburgh Sleep Quality Index and overweight and obesity in youth (23). Poorer self-reported sleep quality, defined as higher sleep onset latency, more sleep disturbances, recurrent awakenings, and lower sleep efficiency, was associated with a higher odds of being overweight or obese (odds ratio of 1.46, 95% CI of 1.24–1.72), independent of sleep duration.

However, some studies have not found an association between sleep quality measures aside from sleep duration and weight status in younger children (24–26). When sleep quality measures of sleep latency, wake after sleep onset, sleep duration, and sleep efficiency were collected using a parental-reported sleep diary and objectively using wrist actigraphy, no association was found between sleep quality and body mass index, body fat percentage, or waist circumference (26). Of note, there was a low prevalence of overweight and obese subjects in this study, with only 7% having a BMI in this range.

## Sleep chronotype and obesity

Adolescent preferred sleep patterns lead to decreased sleep times, both because their biologic circadian rhythm shifts toward later sleep and wake times resulting in a “late chronotype” and because of competing interests to complete schoolwork and socialize during evening and nighttime hours (3). Chronotype is the sleep timing of an individual or that individual's propensity to sleep during a particular time. Early adolescence and puberty are biologically linked to later bed time and wake time preferences, known as an evening-type circadian rhythm or evening chronotype. Pubertal adolescents and young adults have a slower escalation in “drive-to-sleep” or lower sleep pressure, compared with their prepubertal counterparts. As sleep onset is delayed by both biological and societal influences and wake times tend to be fixed, sleep deprivation accumulates.

“Bedtime shift” describes later bed times on weekends compared to weekdays and may also affect the circadian rhythm as the circadian clock cannot adapt quickly to shifts in sleep onset. Independent of sleep duration, greater bedtime shift is linked to obesity severity (14). Circadian rhythm disorder or delayed sleep-phase syndrome is associated with daytime sleepiness and can lead to napping after school, ending ultimately with difficulty sleeping at night. In 186 adolescents, ages 12–17 years, sleep quality assessed using the self-reported Insomnia Severity Index, was not related to a higher BMI z-score (14). However, there was a significant association between a higher BMI z-score and a later weekend bedtime, and a greater bedtime shift. Ivers-Landis et al. evaluated self and parent-reported dietary, sleep duration, and sleep regularity parameters (bedtime shift, wake-time shift, and sleep duration shift) among 315 overweight and obese adolescents (27). Late bedtime shift and wake-time shift were associated with increased sugar-sweetened beverage consumption and food preoccupation.

Both biological influence and social influences that encourage later sleep times and decreased sleep duration are associated with obesity. Social jetlag describes the alterations in one's chronotype due to social obligations such as school, work, or other social events. Roenneberg et al. assessed social jetlag and obesity in a database of 64,110 primarily central European subjects, ages 16–65 years, who had completed the Munich ChronoType Questionnaire (28). Social jetlag, defined as the difference between mid-sleep point on free days vs. work days, significantly increased the odds of being overweight by 3.3 (95% CI 2.512–4.334). The association between a late chronotype and BMI was studied in 511 young adolescents, ages 11–13 years, in the United Kingdom by Arora and Taheri in a cross-sectional study (29). A definitely evening chronotype, identified by the lowest score category on the self-reported Morningness Eveningness Questionnaire, represented 15.3% of the sample size and was positively associated with BMI z-score (*p* < 0.01).

Hulsegge et al. compared diet quality and quantity between 7,173 adult day workers and 683 adult shift workers using cross-sectional general population data from the European Prospective Investigation into Cancer and Nutrition-Netherlands cohort (30). Dietary intake was assessed using a food frequency questionnaire and dietary quality was assessed using the Mediterranean Diet Score and WHO-based Healthy Diet Indicator. Although shift workers had a similar diet quality to day workers, their energy intake was higher by 56 kcal/day (95% CI of 10–101). Shift workers with five or more night shifts per month had the highest energy intake, taking in an additional 103 kcal/day (95% CI of 29–176) than day workers consumed. These results suggest high energy intake may contribute to the metabolic disturbances (30), such as increased body weight, elevated systolic blood pressures, dysglycemia, type 2 diabetes, dyslipidemia, and cardiovascular disease, which have been linked with shift work (31).

## Sleep, glucose metabolism, and cardiometabolic risk

Current evidence indicates sleep disturbances contribute to alternations in glucose metabolism and increased cardiometabolic risk (32–34). Similar to adults, poor self-reported sleep quality in youth has been associated with components of the metabolic syndrome, including dyslipidemia, higher blood pressure, and markers of insulin resistance (35–39). Alterations in leptin, ghrelin, and cortisol levels and increased sympathetic nervous system activity are thought to contribute to an atherogenic lipid profile (40). Qian et al. proposed that sleep fragmentation alters lipid metabolism via its effect on elevated cortisol levels, increased systemic inflammation, increased food intake, and obesity (41). Increased sympathetic nervous system activity secondary to insufficient sleep contributes to hypertension (38). Moreover, deficient deeper stages of sleep (REM and slow-wave sleep) are associated with an elevated morning blood pressure in obese adolescents, independent of weight status (36). If poor sleep is contributing to elevated blood pressures and metabolic syndrome in youth, interventions should be pursued, as the cumulative effects of these risk factors in obese youth are significant.

In 2016, the first study was published that investigated the association between sleep deprivation and insulin sensitivity performed via hyperglycemic clamp in 81 adolescents (32). Those with sleep deprivation, defined as less than 8 h of sleep per night, had a lower clamp-derived insulin sensitivity index, compared to those with sufficient sleep, defined as 8 h or more.

Other pediatric studies indicate obese children and adolescents who report less than 9 h of sleep per night have higher fasting insulin and HOMA-IR levels, and lower high-density lipoprotein cholesterol (HDL-C) levels (42). Interestingly, a U-shaped distribution was observed, as those with intermediate sleep durations, 9–10 h for 10–13 year olds and 8–9 h for 14–15 year olds, had the lowest HOMA-IR, higher HDL-C, and lower aspartate aminotransferase (AST). Those with higher or lower sleep durations had higher HOMA-IR consistent with greater insulin resistance. This U-shaped distribution has been demonstrated for sleep duration and hemoglobin A1c (HbA1c) and glucose levels during an oral glucose tolerance test (OGTT) as well (33, 43).

## Obstructive sleep apnea in obese adults with type 2 diabetes

A strong association between type 2 diabetes and OSA is seen in overweight and obese adults (44). The Sleep AHEAD (Action for Health in Diabetes) Study is an ancillary study measuring the prevalence of OSA in participants in the Look AHEAD study, a 16-center prospective trial examining the effects of an intensive lifestyle program in overweight and obese adults with type 2 diabetes (44). The Sleep AHEAD study demonstrated an alarmingly high prevalence of undiagnosed OSA in this population at 86% and an elevated average apnea-hypopnea index (AHI) of 20.5 events per hour. The only significant predictor of OSA was a higher waist circumference, but subjects with a higher BMI were more likely to have severe OSA (44). A meta-analysis of prospective cohort studies to evaluate the association between OSA severity and the risk for type 2 diabetes was performed by Wang et al. and included 6 prospective studies with almost 6,000 adult patients (45). Included studies used only objective measurements for the diagnosis of OSA and found moderate (AHI 15 to less than 30) and severe OSA (AHI 30 or greater) was an independent risk factor for type 2 diabetes in adults. Another meta-analysis of prospective and retrospective cohort studies in adults to assess the association between OSA and metabolic syndrome involved 10 studies with just over 2,000 patients (46). This meta-analysis revealed OSA was a significant risk factor for components of the metabolic syndrome, specifically higher systolic blood pressure, lower HDL, higher LDL (low-density lipoprotein), and higher triglyceride levels.

Importantly, the Sleep AHEAD study focused on the effect of weight loss on OSA (47). The two intervention groups in Look AHEAD and Sleep AHEAD are Intensive Lifestyle Intervention (ILI) and Diabetes Support and Education (DSE). ILI gives participants specific and detailed recommendations for portion control, daily caloric intake limits, and a physical activity requirement. Whereas DSE provides education and support on diet, physical activity, and social support, but without specific behavioral strategies (47, 48). Studies looking at the effects of weight loss on OSA in obese adults with type 2 diabetes are promising for the ILI group, as this group lost significantly more weight and had a significant decrease in the AHI after 1 year (48). At 1 year, three times more participants in the ILI than the DSE group had total OSA remission and severe OSA prevalence was halved in the ILI group. This effect of improved AHI in the ILI group was sustained even over the long-term period of 4 years despite a 50% regain in weight, suggesting the benefits of this program are more than just weight loss alone (47).

Proposed mechanisms for an association between OSA and dysglycemia are multifactorial. OSA can lead to activation of the sympathetic nervous system, increased leptin and ghrelin levels contributing to increased appetite and food intake, decreased adiponectin level, oxidative stress, inflammation and obesity which all ultimately contribute to insulin resistance (49, 50). The relationship between OSA and type 2 diabetes may be explained by the stage of sleep when apneas and hypopneas are occurring, as these episodes are longer and have greater oxygen desaturations during REM vs. non-REM sleep, subsequently leading to greater sympathetic nervous system activity (51). These alterations may have an effect on insulin release and activity as an altered sympathetic/parasympathetic balance may affect hormones involved in glucose regulation (52). In a cohort of 115 adult subjects, only the REM AHI was associated with an increased HbA1c (52).

## Obesity and metabolic consequences of OSA in youth

In youth, the association between OSA and type 2 diabetes is less clear (33). Previous studies have shown inconsistencies across studies in youth, with small sample sizes and pubertal effects on insulin sensitivity affecting measures. A recent systematic review and meta-analysis of 10 studies by Patinkin et al. attempted to clarify relationships between OSA and metabolic risk markers in youth by focusing on studies with exclusively adolescent participants (53). OSA in adolescence was associated with dyslipidemia, hypertension, and insulin resistance as measured by HOMA-IR, as in adults.

A few pediatric studies have evaluated OSA and glucose homeostasis in obese youth (54–57) and have provided preliminary evidence of an association between OSA and lower insulin sensitivity with increased fasting plasma insulin and glucose levels, independent of BMI (33). Despite similar degrees of obesity, adolescents with moderate or severe OSA (AHI 5 or greater), as opposed to mild or no OSA, had significantly higher HOMA-IR (*p* = 0.0497) and fasting insulin levels (*p* = 0.037) (58). Redline et al. reported a cohort study in which 70% of adolescents with sleep-disordered breathing (SDB) were overweight and 59% of them met criteria for metabolic syndrome, notably elevated blood pressure, LDL, and fasting insulin levels, again independent of BMI (57). Only 16% of overweight adolescents without SDB had metabolic syndrome and after adjustment for age, race, sex, and prematurity, adolescents with SDB had an increased odds of 6.49 (95% CI 2.52–16.70) for metabolic syndrome compared to overweight adolescents without SDB.

A study from Beijing, involving 558 participants ages 14–28 years, demonstrated that even youth with high-risk for OSA measured by self-reported Berlin Questionnaire score had higher cardiometabolic risk including dyslipidemia, higher glucose levels during OGTT, elevated liver enzymes, non-alcoholic fatty liver disease, metabolic syndrome, and worse echocardiographic parameters, specifically higher interventricular septum thickness, left ventricular end diastolic diameter, and left ventricular posterior wall thickness (59).

However, there are also pediatric studies which have not reported a link between OSA and increased markers of metabolic risk. Erdim et al. did not find an association between OSA measured with overnight polysomnography and metabolic syndrome in 104 obese adolescents (60). AHI was 1 or greater in 47.2% of youth without metabolic syndrome and in 49% of youth with metabolic syndrome. In an attempt to focus on the association of metabolic syndrome and OSA in obese adolescents by minimizing confounding factors, they uniquely excluded patients with grade 3 or 4 adenoidal or tonsillar hypertrophy, a known common cause of OSA. Narang et al. found that intermittent nocturnal hypoxia, not OSA measured by AHI was associated with a higher fasting insulin level, more insulin resistance as measured by HOMA-IR, higher HbA1c, and increased AST and ALT after correction for waist-to-height ratio (61). These studies used different criteria for evaluating metabolic risk and therefore, larger studies with uniform outcome measures are needed.

A prospective, cross-sectional study by Hannon et al. of 57 obese adolescents with either normal glucose tolerance, dysglycemia, or type 2 diabetes showed those with dysglycemia or type 2 diabetes tended to have a higher AHI, but no linear relationship between glycemia and AHI was established (62). Another prospective, cross-sectional study by Shalitin et al. compared 11 obese youth with type 2 diabetes compared to 30 obese youth without diabetes who were BMI-SDS matched in order to evaluate the frequency and severity of OSA between these groups (63). The age range of youth for both groups was 6–21 years and mild OSA was defined as an AHI greater than 1, while moderate to severe OSA was defined as an AHI 5 or greater. There was no significant difference in the frequency or severity of OSA between obese patients with type 2 diabetes and those with normoglycemia. The power of these relatively small studies to detect a significant association has been limited. Nevertheless, if there is an association between OSA and poor glycemic outcomes and type 2 diabetes in youth, this would be important for intervention and prevention efforts in these obese youth. This calls for more support for large pediatric studies to clearly delineate the association between OSA and dysglycemia in obese youth. Table 2 includes suggested areas in need of further research, especially in youth.

**Table 2:** Areas for future research in sleep and obesity in youth.

Sleep and Dyslipidemia Mechanisms to Determine Causality between Sleep and Obesity Chronotype and Obesity Obesity and risk for Type 2 Diabetes OSA and risk for Type 2 Diabetes Sleep Interventions to Improve Obesity and Cardiometabolic Risk

## OSA and treatment effect

A systematic review of outcomes of OSA treatment in obese youth showed that obese children are significantly more likely to have persistent OSA after adenotonsillectomy than normal-weight youth and consistent with results in adults, either behavior or surgical weight loss, significantly improves OSA in obese youth (64). However, studies are few and laden with limitations, demonstrating the need for more research into weight loss as a treatment option. Positive airway pressure is also effective, but adherence is a major challenge (64). A systematic review and meta-analysis of six studies assessed the effects of continuous positive airway pressure (CPAP) in adult patients with type 2 diabetes and OSA (65). Results showed improved insulin sensitivity, but no decrease in BMI or HbA1c level with at least 3 months of CPAP. Another adult study summarizing three randomized controlled trials with 149 total patients showed that CPAP withdrawal in patients with optimal compliance was associated with a return of OSA and clinically significant increases in blood pressure (66). In the CPAP withdrawal group compared to the CPAP continuation group, office systolic blood pressure, home systolic blood pressure, office diastolic blood pressure, and home diastolic blood pressure all significantly increased. In adults, improved CPAP adherence that involves more coverage of REM sleep, can improve HbA1c (51, 52).

## Sleep interventions and health in youth

Although the association between poor sleep and obesity is clear, causation has yet to be determined. Preliminary studies in youth show some evidence that improved sleep may improve obesity risk (67). In fact, interventions such as earlier bedtimes, later wake times, and other healthy sleep behaviors could be low-cost (9). A pilot study using a sleep hygiene program involving 33 adolescents showed improvement in self-reported sleep quality and BMI z-scores, but not sleep duration measured by actigraphy (68). Sleep quality was measured using participant or parent questionnaires (Adolescent Sleep Hygiene Scale, Pittsburgh Sleepiness Scale, Sleep Disturbance Scale for Children, and Pediatric Daytime Sleepiness Scale). BMI z-scores decreased significantly from a baseline mean of 0.79 (*SD* 1.18) to a post-intervention mean of 0.66 (*SD* 1.19). While preliminary studies are showing potential, well-designed randomized controlled trials to assess if sleep interventions ultimately improve obesity and cardiometabolic health in youth are warranted.

## Conclusion

Poor sleep and obesity are concurrent epidemics in youth. Insufficient sleep duration and poorer sleep quality are associated with a greater BMI and markers of cardiometabolic risk, including insulin resistance, dyslipidemia, and higher blood pressure in youth. Obese youth are more likely to have OSA linked with cardiometabolic risk, but a clear association between OSA and type 2 diabetes in youth has not been established. Whether sleep interventions can improve pediatric obesity and cardiometabolic health in youth is yet to be determined.

## Author contributions

AG and TH contributed to concept and outline of the manuscript. AG wrote the first draft and sections of the manuscript. TH revised manuscript critically for important intellectual content. AG and TH contributed to manuscript revision, read, and approved submitted version. AG and TH agree to be accountable for all aspects of the work.

### Conflict of interest statement

The authors declare that the research was conducted in the absence of any commercial or financial relationships that could be construed as a potential conflict of interest. The reviewer KO and handling Editor declared their shared affiliation.
